# *Eurosurveillance* reviewers in 2019

**DOI:** 10.2807/1560-7917.ES.2020.25.1.2001092

**Published:** 2020-01-09

**Authors:** Eurosurveillance editorial team

**Affiliations:** 1 European Centre for Disease Prevention and Control (ECDC), Stockholm, Sweden

**Keywords:** peer-review

The editorial team is grateful to all the experts who dedicated their time to peer-review our articles over the past 12 months. We wish to thank them and all those whose names are not listed below but gave helpful advice and support. We apologise to any reviewers whose names we may have missed.

Our reviewers in 2019 were:

Sam Abbott; Stephan Aberle; Amos Adler; Cornelia Adlhoch; Karoline Aebi-Popp; Anton Aebischer; Seven Johannes Sam Aghdassi; Franz Allerberger; Erik Alm; Katharina Alpers; Christian Althaus; Laura Anderson; Nick Andrews; Andrea Angheben; Muna Anjum; Markus Antwerpen; Kevin Ariën; Wouter Arrazola de Oñate; Mardjan Arvand; Thomas Atkinson; Agoritsa Baka; Vincenzo Baldo; Eszter Balla; April Baller; Mathieu Bangert; Fernardo Baquero; Ian Barr; Luisa Barzon; Ulrike Baum; Julien Beauté; Ron Behrens; Antonino Bella; Edward Belongia; Kimberley Benschop; Dennis Bente; Bojana Beović; Béatrice Berçot; Juan Berenguer; Christoph Berger; Claire Bertelli; Sophie Bertrand; Marianne Besnard; Debra Bessen; Cornelia Betsch; Yunus Emre Beyhan; Julia Bitzegeio; Carina Blackmore; Amandine Boeuf; Shelly Bolotin; Raquel Bonelli; Marc Bonten; Michael Borg; João Botelho; Hervé Bourhy; Martijn Bouwknegt; Naomi Boxall; Marieta Braks; Viviane Bremer; Deborah Briggs; Folke Brinkmann; Eeva Broberg; Tal Brosh-Nissimov; Julia Brotherton; Colin Brown; Springer Browne; Mia Brytting; Jacqueline Bua; Silke Buda; Susanne buder; Niccolò Buetti; Muruleedhara Byappanahalli; Sindy Böttcher; Saverio Caini; Paolo Calistri; Christine Campese; Rosa Cano; Catherine Carillo; Yehuda Carmeli; AnnaSara Carnahan; João André Carriço; Otto Cars; Jordi Casabona; Jean-Sebastien Casalegno; Alessandro Cassini; Francesco Castelli; Jesus Castilla Catalan; Vincent Cattoir; Dominique Caugant; Matthias Cavassini; Remi Charrel; Anuja Chatterjee; Didier Che; Daniel Chemtob; Yu Chen; Tom Chiller; Sadegh Chinikar; Gerardo Chowell; Iva Christova; Nadia Ciampa; Tristan Clark; John Coia; Rosalind Lucy Coleman; Vittoria Colizza; Sarah Collier; Deirdre Collins; Alex Cook; Michael Cooper; Vicente Corrales-Medina; Andrea Cortegiani; Benjamin Cowling; Amelieke Cremers; Sara Croxford; Gabriela da Silva; Ron Dagan; Eilif Dahl; Richard Danila; Eric Dannaoui; Mark Danta; Nicolas Dauby; Helena de Carvalho Gomes; Alexis de Rougemont; Gaston De Serres; Jaime Delgado; Valerie Delpech; Sarika Desai; Antonino Di Caro; Liselotte Diaz Högberg; Julia Dina; Steven Djordjevic; Gerhard Dobler; Roger Dodd; Margaret Doll; Pierre Dorny; Jan Drexler; Christian Drosten; Matthew Dryden; Sandra Dudareva-Vizule; Erika Duffell; Tim Eckmanns; Adrian Egli; Anna-Maria Eis-Huebinger; Andreas Halgreen Eiset; Joanna Ellis; Theresa Enkirch; Olivier Epaulard; Connie Erkens; Sylvie Escolano; Steen Ethelberg; Matthieu Eveillard; Mirko Faber; Dennis Falzon; Cecilia Fazio; Vincenzo Ferrantelli; Lena Fiebig; Nigel Field; Helen Fifer; Marc Fischer; Thea Fischer; David Fisman; Brendan Flannery; Gilles Folléa; Anders Fomsgaard; Kevin Fonseca; Ron Fouchier; Christina Frank; Hans Fredlund; David Freedman; Alexander Friedrich; Ingrid Friesema; Angelika Fruth; Wakaba Fukushima; Luis Furuya-Kanamori; Eric Fèvre; Giovanni Gabutti; Arnaud Gagneur; Wolfgang Gaissmaier; Juan Carlos Galan; Ray Gamble; Sumanth Gandra; Giuliano Garofolo; Petra Gastmeier; Philippe Gautret; Peter Gerner-Smidt; Anne Gershon; Monica Gianfranceschi; Noel Gill; Aharona Glatman-Freedman; Youri Glupczynski; Judith Glynn; Sonia Gomez; Katie Goodman; Magnus Gottfredsson; David Graham; Marc Grandadam; Sophie Granier; Stefan Gravenstein; Christina Greenaway; Gilbert Greub; Emma Griffiths; Thomas Grönthal; Jonathan Gubbay; Nicole Guiso; Jean-Paul Guthmann; Jennifer Guthrie; Bernardo Rafael Guzman Herrador; Stephan Günther; Bart Haagmans; Christos Hadjichristodoulou; Sebastian Haller; Anette Hammerum; Mohamed Hammoud; Dag Harmsen; Heli Harvala; Amber Haynes; John P Hays; Jeanne Heil; Marianne Heisz; Bonnie Henry; Niel Hens; Victoria Hernando; Bjorn Herrmann; Roger Hewson; Chelsea Himsworth; Andy Hjortenfeldt; Michele Hlavsa; Heidemarie Holzmann; Yvan Hutin; Benedikt Huttner; Václav Hönig; Judith Hübschen; Greet Ieven; Derval Igoe; Giuseppe Indolfi; Naoki Inoue; Giuseppe Ippolito; Charlotte Jackson; Michael Jackson; Yves Jackson; Kathryn Jacobsen; Marie Jansson Mork; Meryem Jefferies; Claire Jenkins; Seok Hoon Jeong; Cecilia Jernberg; Dakshika Jeyaratnam; Alan Johnson; Jerker Jonsson; Tsutomu Kageyama; Alexander Kallen; Anu Kantele; Tommi Karki; Basel Karo; Mark Katz; Alyson Kelvin; Yoav Keynan; Hirokazu Kimura; Pete Kinross; Martyn Kirk; Irena Klavs; RM Klevens; Don Klinkenberg; Mirjam Knol; Anke Kohlenberg; Jeroen Kortekaas; Maria Korzeniewska-Koseła; Rolf Kramer; Tyra Krause; Peter Kreidl; Lothar Kreienbrock; Pavla Krizova; Manuel Krone; Yao Kudjawu; EJ Kuijper; Jaana Kusnetsov; Jeff Kwong; Sharon Kühlmann-Berenzon; Angie Lackenby; Anna Ladogana; Iain Lake; Theresa Lamagni; Sarah Larney; Amparo Larrauri; Heidi Larson; Jeffrey Lazarus; Simon Le Hello; Alexandre Leclercq; Sylvie LeCollinet; Philippe Lehours; Andreia Leite; Katrin Leitmeyer; Isabelle Leparc-Goffart; Tinne Lernout; Daniel Levy-Brühl; Monica Licker; Diane Lindsay; Theodore Liou; Luiz Lisboa; Nicola Low; Claudia Lucarelli; Catherine Ludden; Christian Lueck; Alexander Lukashev; Åke Lundkvist; Irja Lutsar; Theodore Lytras; Kristine Macartney; Emily MacDonald; Anna Machowska; Shabir Madhi; Kirsten Maertens; Alexandra Mailles; Helena Maltezou; Georgia Mandilara; Antonella Marangoni; David Marchant; Ulrich Marcus; Giovanni Marini; Robby Markwart; Emily Martin; Helena Martini; Federico Martinón-Torres; Josefa Masa-Calles; Alexander Mathis; Peter McIntyre; Jim McMenamin; Paul S. Mead; Adam Meijer; Wim Meijer; Jacques Meis; Hasse Melbye; Tanya Melillo Fenech; Stefano Merler; João Mesquita; Peter Michielsen; Sofie Midgley; massimo mirandola; Vivi Miriagou; Thomas Mollet; Dominique Monnet; Ginny Moore; Anne Moorman; Orna Mor; Alain Moren; Pedro l. Moro; Lapo Mughini-Gras; Otilia Mårdh; Kåre Mølbak; Marcel Müller; Anthony Nardone; Umaer Naseer; Pontus Naucler; Nichola Naylor; Ndeindo Ndeikoundam Ngangro; Laura Nellums; Martha Nelson; Nathalie Nicolay; Hubert Niesters; Hiroshi Nishiura; Karsten Noeckler; Harold Noel; Hanna Nohynek; Monika Novak Babic; Sofia Ny; Akihiro Ohkado; George Okoli; Isabel Oliver; Orjan Olsvik; Ryosuke Omori; Nitika Pai; Annalisa Pantosti; Anna Papa; Constantinos Papagiannitsis; John Papp; Dimitrios Paraskevis; Maria Pardos de la Gandara; Sydel Parikh; Maria Paulke-Korinek; Richard Pebody; Tatjana Pekmezovic; Pasi Penttinen; Jose Perez-Molina; Tom Peterman; Efi Petinaki; Martin Petric; Joshua Petrie; Michael Pfaller; Dina Pfeifer; Diamantis Plachouras; Leo Pomar; Stefano Pongolini; Lia Possuelo; Spyridon Pournaras; Edoardo Pozio; Namrata Prasad; Fabian Prasser; Elisabeth Presterl; Jan Prins; Katarina Prosenc; Andrea Pugliese; Joan Puig-Barbera; Mirja Puolakkainen; Mario Ramirez; José Tomás Ramos Amador; Barbara Rath; Pedro Reis Costa; Vincenzo Restivo; Chantal Reusken; Juliana Reyes Urueña; Claire Reynolds; Giovanni Rezza; Flavia Riccardo; Jean-luc Richard; Hans Rieder; Shmuel Rishpon; Caterina Rizzo; Lucy Robertson; Emmanuel Robesyn; Eve Robinson; Susie Roczo-Farkas; Ana Rodrigues; Melissa Rolfes; Magdalena Rosinska; Mirko Rossi; Adam Roth; Markus Ruhnke; Werner Ruppitsch; Daniel Ruzek; Helio Sader; Luisa Salazar; Henrik Salje; Dan Salmon; Vittorio Sambri; Andre Sasse; Gianpaolo Scalia-Tomba; Franciska Schets; Patricia Schlagenhauf; Daniela Schmid; Jonas Schmidt-Chanasit; Isabelle Schuffenecker; Eli Schwartz; Kevin Schwartz; Holly Seale; Harald Seifert; Aaron Seigler; Makeda Semret; Nariman Shahhosseini; Jingjing Shang; Giri Shankar; David Shay; Annette Siedler; Jonne Sikkens; Ian Simms; Benedetto Simone; yannick simonin; Nalini Singh; Vinciane Sizaire; Danuta Skowronski; Rami Sommerstein; Klara Sondén; Carla A. Sousa; Ian Spicknall; Gianfranco Spiteri; Marc Steben; Anneke Steens; J Stelling; Andrew Stewardson; Reinhild Strauss; P Strebel; James Stuart; Bertrand Sudre; Carl Suetens; Sheena Sullivan; Lawrence Svenson; Eduardo Taboada; Emi Takashita; Johanna Takkinen; Lara Tavoschi; Nasrin Tayyari Dehbarez; Anne Teirlinck; Laura Temime; Tristan Timbrook; Giota Touloumi; Ramona Trebbien; Athanasios Tsakris; Sarah Tschudin-Sutter; Rainer Ulrich; Anthony Underwood; Magnus Unemo; Tim Uyeki; Marta Valenciano; Palle Valentiner-Branth; Marti Vall-Mayans; Birgit van Benthem; Marita van de Laar; Mark van der Linden; Nicoline van der Maas; Marianne van der Sande; Marieke J. van der Werf; Sonja van Roeden; Ivo van Walle; David Vanduin; Philippe Vanhems; Daisy Vanrompay; Olli Vapalahti; Paula Vasconcelos; Nikos Vasilakis; Alkiviadis Vatopoulos; Jaap Veen; Tomas Vega; Aurelie Velay; Inga Velicko; Akke Vellinga; Harry Vennema; Giulietta Venturi; Lasse Vestergaard; Mafalda Viana; Antonio Vieira; Tatjana Vilibic-Cavlek; Jan Vinjé; Gianluca Voglino; Alex Vorsters; Georgia Vourli; Tim Wade; Maryse Wanlin; Mary Warrell; Charlotte Warren-Gash; Keith Warriner; Don Weiss; Herbert Weissenboeck; Klaus Weist; Dirk Werber; Guido Werner; Therese Westrell; David Whiley; Heather Whitaker; Ursula Wiedermann; Annelies Wilder-Smith; Hendrik Wilking; Krista Wilkinson; Julie Wilson; Marc Wirden; Carl-Heinz Wirsing von König; Martin Wolkewitz; Ian Woolley; Zoe Yandle; Lin Yang; Fathiah Zakham; Peter Zarb; Walter Zingg; Milan Čižman.

